# Using Ecological Momentary Assessment to Evaluate Current Physical Activity

**DOI:** 10.1155/2014/915172

**Published:** 2014-07-14

**Authors:** Jolanta Marszalek, Natalia Morgulec-Adamowicz, Izabela Rutkowska, Andrzej Kosmol

**Affiliations:** Faculty of Rehabilitation, Józef Piłsudski University of Physical Education in Warsaw, ul Marymoncka 34, 01-813 Warsaw, Poland

## Abstract

*Objective*. The purpose of this study was to assess the value of ecological momentary assessment in evaluating physical activity among children, adolescents, and adults. It also determines whether ecological momentary assessment fulfills the criteria of validity, reliability, objectivity, norms, and standardization applied to the tools used for the evaluation of physical activity. *Methods*. The EBSCO*-*CINHAL, Medline, PsycINFO, PubMed, and SPORTDiscuss databases were reviewed in December 2012 for articles associated with EMA. *Results*. Of the 20 articles examined, half (10) used electronic methods for data collection, although various methods were used, ranging from pen and paper to smartphone applications. Ten studies used objective monitoring equipment. Nineteen studies were performed over 4 days. While the validity of the EMA method was discussed in 18 studies, only four found it to be objective. In all cases, the EMA procedures were precisely documented and confirmed to be feasible. *Conclusions*. Ecological momentary assessment is a valid, reliable, and feasible approach to evaluate activity and sedentary behavior. Researchers should be aware that while ecological momentary assessment offers many benefits, it simultaneously imposes many limitations which should be considered when studying physical activity.

## 1. Introduction

Physical activity (PA) is understood in many ways. Caspersen et al. [1] define PA as “any bodily movement produced by skeletal muscles which results in energy expenditure” [1, 2]. However, another definition, accepted by social scientists, states that PA is “behavior which can be conceptualized on a continuum from minimal to maximal movement” [3]. The term “exercise,” defined as “a subset of PA that is planned, structured, and repetitive bodily movement done in order to improve or maintain one or more physical components of physical fitness,” is also frequently used [3] and accepted by the American College of Sports Medicine (ACSM).

In addition to PA, two other terms are often defined while setting reference standards or benchmarks for children and adolescents: “physical inactivity” and “sedentary behavior.” In children, physical inactivity is defined as a “PA level that is lower than in healthy individuals of similar age, gender, and cultural and socioeconomic background” [3]. A sedentary lifestyle is described by the ACSM as “not participating in a regular exercise program or not meeting the minimal PA recommendations” [3]. Marshall and Welk argue that an insufficient level of PA is not physical inactivity but “sedentary behavior” [3].

Physical activity (PA) can be assessed by objective and subjective methods, and these should fulfill several eligibility criteria: validity, reliability, objectivity, norms, and standardization [4, 5]. Objective methods are based on mechanical/electronic monitoring using accelerometers, pedometers, or heart rate monitors or on the measurement of such physiological parameters as direct and indirect calorimetry, maximum oxygen consumption/VO_2_max, doubly-labeled water consumption, or energy expenditure. Alternatively, subjective methods based on direct observation or questionnaires can be used [2, 3], and these can be applied to the assessment of sedentary behavior. The strength of the questionnaire method is its ability to assess the type, location, and prevalence of PA, for example, whether it occurs in school or during free time activities, with whom PA is undertaken, for example, with friends, family, or alone, and the emotions associated with PA. Furthermore, as objective methods do not differentiate between periods of inactivity and periods when the device is not being worn, subjective methods are preferred for measuring sedentary behavior [3]. Within the group of subjective methods, the “daily life” sampling approaches, such as the experience sampling method (ESM) and ecological momentary assessment (EMA), have recently aroused considerable scientific attention [6]. Some authors use the terms ESM and EMA interchangeably but Trull and Ebner-Priemer [6] emphasize that there are two different methods. EMA is viewed as a broader methodology that attempts to integrate a number of assessment traditions of self-reported subjective states and behavior, including ESM, [7] with the sampling and monitoring of physiological processes, behaviors, or states by electronic devices such as mobile phones [6].

In the fields of rehabilitation, sport science, and behavioral medicine, EMA has often been used to assess the dynamic changes in the behavior of children, young people, and adults, based on their PA and sedentary behavior [8]. Not only does it allow data to be collected in real time [9], but also the clinical and theoretical complexity of PA can be examined in a real-world environment, along with its associated problems and dynamic processes [8]. EMA analysis highlights individual differences in behavior, their distribution over time, the factors affecting their behavior, and the mutual associations between them. A comprehensive ecological approach provides rich, diverse, and complex data which can be used in the analysis of behavior [7].

Many studies have described the advantages of using EMA methodology to assess current activity [7, 10–12], the most important one being that the EMA approach combines both ecological and momentary aspects. Its ecological aspect is represented by data collection being performed in the real world, which allows the available information to be generalized to the real life of individuals, hence providing ecological validity. Its momentary aspect is defined by the real-time collection of information, in this case about PA, which minimizes errors caused by the necessity of recalling what had happened in the past. The difficulty of recalling the past often constitutes an obstacle to traditional methods of assessing PA, which usually restricts measurement to intervals of less than one day. However, as information is gathered at random intervals or is selected based on a pilot study during EMA analysis, it is possible to observe behavioral changes and the factors causing such changes, as subjects repeatedly answer the same set of questions during a single study [7, 10–12].

Data can be collected in several ways using the EMA approach, such as questionnaires, diaries, or electronic devices running special software. It can also be collected through “pencil and paper” questionnaires sent and verified each day during an ongoing study [11]. Alternatively, electronic methods such as online questionnaires [13] or portable electronic devices such as PDAs (personal digital assistants) and mobile phones can be used [11, 13–15]. The PDA collects data, informs subjects when which they are supposed to complete the questionnaire, and sends the acquired data directly to the authors [11], while free, easy-to-use software such as MyExperience can be installed on mobile phones [16, 17].

The TelEMA platform, a telephone assessment platform for clinical and research applications, is regarded as an effective method of assessing behavior in real time by EMA. It sends signals and data between the experimenter and the test subject via the telephone network. The sent signal indicates a specific time to report current activity and integrates returning calls or text messages with the EMA survey. Data can be simultaneously collected this way by many researchers working in different locations on different projects [13].

The EMA approach allows data to be collected at regular intervals, such as once per day at a regular time or at random times during the day [7, 11]. If random requests are sent, the total number of samples must be first determined and must be the same for all individuals [11]. The time of data collection may also be chosen depending on the aim of the study, for example, whether it addresses events or behavior which occur during the day or during PA [18].

The purpose of this study was to determine whether EMA is a suitable approach to evaluating physical activity among children, adolescents, and adults. A second aim is to determine whether EMA meets the criteria of measurement tools using to evaluate physical activity: validity, reliability, objectivity, norms, and standardization.

## 2. Method

**Figure 1:**
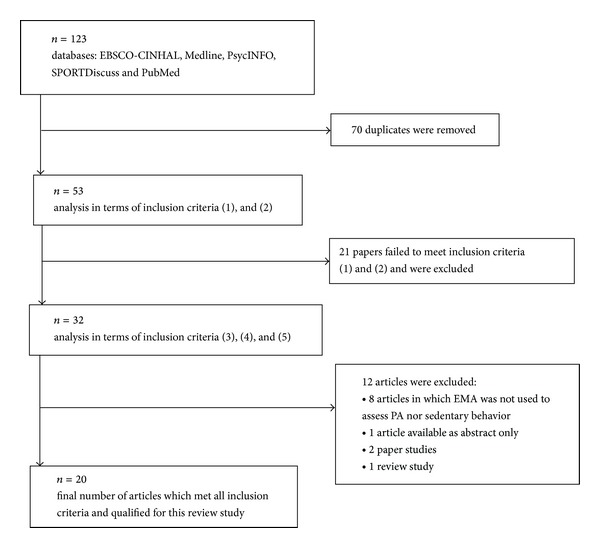
A summary flowchart of the search, selection, and inclusion process. Note: (1) the article had to be available in English, (2) the summary or title of article had to include the following keywords “ecological momentary assessment” and “physical activity” or “ecologic momentary assessment” and “physical activity,” (3) EMA had to be used to evaluate PA and sedentary behavior, (4) the article had to be available as full text, and (5) the article had to be a research study.

The EBSCO-CINHAL, Medline, PsycINFO, PubMed, and SPORTDiscuss databases were reviewed in December 2012. The inclusion criteria of reviews were that (1) the article had to be available in English, (2) the summary or title of the article had to include the following keywords “ecological momentary assessment” and “physical activity” or “ecologic momentary assessment” and “physical activity,” (3) EMA had to be used to evaluate PA and sedentary behavior, (4) the article had to be available as full text, and (5) the article had to be a research study. A summary flowchart of the search, selection, and inclusion process is presented in Figure 1.

## 3. Results

A total of 53 unique articles were found in the EBSCO-CINHAL, Medline, PsycINFO, PubMed, and SPORTDiscuss databases. Thirty-two of these articles met the inclusion criteria in terms of language (1) and content of keywords in the title or summary (2). The keywords “ecological momentary assessment” and “physical activity” or “ecologic momentary assessment” and “physical activity” were found in the titles and summaries of 14 articles and the abstracts of 18 articles. Another 12 articles were rejected because they either did not use EMA to evaluate PA and sedentary behavior (3), were not available as full text (4), or were not research studies (5). In total, twenty articles were included in this review study [9, 17, 19–36]. The oldest article was published in 2005 [9], whereas the newest articles were published in 2011 [17, 27, 28, 36] and 2012 [29–31].

The included articles were divided into two groups. The first comprised 14 articles in which the subjects were children and adolescents (Tables 1 and 2) [9, 19–31]. The second group comprised 6 articles which focused on adults (Table 3) [17, 32–36]. Only one study concerned older people, aged 50 to 76 [33]. Both girls and boys were used as subjects in 8 articles [20, 22–24, 27, 28, 30, 31], men and women in 3 [17, 34, 35], three only used girls [19, 21, 26], three only women [32, 33, 36], and three only boys [9, 25, 29]. No articles concerned men only.

The most common methods used to collect EMA data were traditional pencil and paper diaries [20–25, 32, 34, 35] and mobile phones with MyExperience software [17, 27–31]. However, diaries for handheld computers [9, 19, 33, 36] and telephone conversations were also used [26]. Seven out of 10 studies were based on electronic methods of collecting data from children and adolescents [9, 19, 27–31].

Studies evaluating activity by EMA among children, adolescents, and adults were commonly conducted twice a year for 4 consecutive days [9, 19–22, 24, 25, 29, 30], over three extended weekends [26], or for several consecutive days [17, 23, 27, 28, 31–36]. In this last group, 50% of the studies evaluated PA at random times over 4 consecutive days: from Saturday to Tuesday-1 [17], from Friday to Monday-3 [27, 28, 31] and over 3 weekdays and 1 day over the weekend-1 [23].

Some studies supported the EMA approach by using such objective tools as accelerometers or heart rate monitors to assess activity [9, 17, 19, 26–31]. Another tool was the talk test, which evaluates the intensity of PA [36]. Such additional tools were most commonly used to assess activity of children and adolescents [9, 19, 26–31].

Furthermore, all studies were evaluated with regard to the choice of activity evaluation tool and its measurement characteristics. While the validity of the EMA method was examined in 18 out 20 studies [9, 17, 19–32, 35, 36], the remaining two cases referred to a different study [33, 34]. The reliability of the EMA approach was not mentioned in only one study [26]. The objectivity of the EMA approach was demonstrated in one study [9], whereas another three [19, 22, 26] referred to other articles for analysis. None of the remaining studies mentioned objectivity. Norms were not described in any of the included articles. In all cases, the EMA procedures were precisely documented and confirmed to be feasible (standardization).

## 4. Discussion and Conclusion

The first aim of this study was to investigate the validity of using ecological momentary assessment (EMA) to evaluate physical activity (PA) among children, adolescents, and adults. The review compiled articles ranging from 2005 to 2012. The relatively short time-span and small number of publications related to this problem suggest that research into the use of EMA to evaluate PA among children, adolescents, and adults is at an early stage.

While 14 articles concerned PA and sedentary behavior among children and adolescents, another 6 addressed it use on adults, and only one publication evaluated activity among the elderly. Moreover, data collected electronically as telephone surveys and electronic diaries predominated among studies focused on children and adolescents. The greater prevalence of electronic forms of collecting EMA data among studies addressed at young people could be due to its easiness of use and the lack of resistance associated with the use of modern technology demonstrated by this specific population. Furthermore, keeping electronic diaries or completing EMA questionnaires on handheld computers or phones could be more attractive for young people than the traditional pencil and paper form of data collection. Older people have been found to be reluctant to participate in studies which promoted electronic forms of data collection [37]. However, it should be noted that information and communications technology is becoming an increasingly common mode of data collection in medicine and health promotion, the main reasons being the lower costs of research and treatment and the more widespread availability of such technology [13, 14].

Many studies use objective measurement tools, such as an accelerometer or a heart rate monitor alongside the subjective EMA assessment. By comparing the data recorded using these devices with the EMA data, it was possible to determine the differences and compliances between the two sources and confirm the construct validity of the study [9, 17, 26–31]. Moreover, most studies using objective measurement tools were intended to evaluate the PA among children and adolescents. Hence, most authors identified whether children were experiencing difficulties and wanted to complete EMA surveys at the time they were involved in some form of activity.

Data comparison provided an indication of the intensity of PA undertaken at the time of submitting the EMA surveys. However, some studies note no significant correlation between the number of steps on a pedometer, or moderate-to-vigorous physical activity (MVPA), and the number of survey questions which remained unanswered [30]. This might indicate a lack of precision in determining a connection between the level of activity intensity, the responses of the subject, and time that the signaled questionnaires were submitted. On the other hand, there might simply be no relationship between the MVPA value and the survey responses. The combination of activity assessment using subjective EMA tools and the evaluation of depression, pain, environmental assessment, or the level of activity intensity using other scales or tests allow accompanying moods, stress levels, pain, barriers to undertaking PA, and levels of PA intensity to be explored [34, 36, 38–40].

An interesting aspect of the evaluated studies was the design and duration of their tests. Many studies [9, 19–25, 29, 30] evaluated the activity twice at given intervals, that is, twice a year, usually in early fall and in the spring. Such a methodology, used in studies focused on children and younger subjects, indicates that the authors intended to demonstrate seasonal variation, changes in PA by the study group depending on the time of year. Moreover, the selection of consecutive days for testing, usually encompassing both weekdays and weekends, allowed observers to evaluate weekly fluctuations in PA participation.

The second aim of this study was to analyze the employed EMA tools in terms of their measurement characteristics. The findings demonstrate that the EMA approach constitutes a valid, reliable, and feasible measurement tool, which clearly indicates that EMA can be considered a suitable method for assessing PA among children, adolescents, and adults.

One of the benefits of using EMA to assess PA among groups of children, adolescents, and adults is that it allows the researcher to gain an insight into factors associated with the activity or behavior itself. Heron and Smyth (2010) [12] emphasize the benefits of the EMA approach. They note that as data collection takes place either at a specific time or during an activity of interest to researchers, the measurement is free from the defects associated with the need for the respondent to remember past events. A second, equally important value of the EMA approach is its ecological validity, that is, that its results can be generalized by its ability to perform measurements in the real world: the authentic surroundings of the respondents. Another advantage is its ability to measure several factors at the same time, for example, PA and the accompanying mood [34], which provides researchers with an opportunity to conduct detailed and more comprehensive examinations, such as identifying relationships between common activities and behavior. These advantages of using EMA to assess PA and sedentary behavior were noted in all studies analyzed in the course of this paper.

There are, however, some limitations associated with EMA and the studies which use it to evaluate PA. A major difficulty is associated with the respondent providing answers in pencil and paper form while involved in PA, particularly when it is intense [9, 19, 30, 31], and more generally, EMA is considered a more burdening and time-consuming approach for participants in comparison with retrospective methods, despite the potential benefits of real-time activity examination [25]. Moreover, adolescents were often found to be reluctant to complete a signaled EMA survey, due to reasons such as a chaotic environment during the survey or the presence of psychological or behavioral problems [9, 19]. Another limitation reported by researchers is the inability to compare EMA results with metabolic energy expenditure, such as kilocalories or metabolic equivalent [9, 20–25, 32–35]. In addition, some studies used EMA only to determine the frequency and duration of the activity, which was considered by some researchers as a limitation of daily PA assessment [19, 28, 30, 31]. Finally, one weakness possessed by all analyzed studies was either an imprecise definition of PA or its absence; in some cases, such activities as active transport to and from school, or activities during school hours, were not taken into account while assessing PA [28, 31].

Furthermore, key similarities and differences exist between the traditional and EMA approaches to assess PA. With regard to the similarities, it is possible to use the same forms of data collection, such as questionnaires or diaries, in both cases [2, 11, 41], and in both cases, subjects may make mistakes while submitting their answers through study protocols [9, 19, 30, 31, 41]. Both EMA and the traditional approach can also be burdening and demanding for the respondents in terms of the time and effort needed to provide required information [25, 41]. However, the obvious benefit of EMA is its ability to collect data in real-time and real-world circumstances [7, 10–12]. The traditional approach to assess PA requires the subjects to recall specific things that occurred at specific times in the past, which may result in inconsistencies and errors in provided information [7, 10–12, 41].

Finally, it should be noted that EMA is coherent with the ecological task analysis (ETA) described by Davis and Burton (1991) [42] and is widespread in the area of adapted physical activity (APA). It has been mentioned that EMA allows the PA profiles of children, adolescents, and adults to be assessed in real-world circumstances and either at specific or at recurring points in time [7, 10–12]. As ETA is a model which considers complex relationships between the individual, the environment, and the task and is used as a system of evaluation and instruction [43], a combination of EMA and ETA may allow the factors inducing engagement in PA to be determined, as well as the influence of environmental limitations on participation in PA. In addition, an ecological approach, taken by both EMA and ETA, allows affective mechanisms and cognitive abilities, or psychomotor skills that have a significant impact on engaging in PA, to be analyzed.

The use of EMA, in its various forms, to assess PA and sedentary behavior is a valid, reliable, and feasible method of evaluation. However, in the assessment of PA, it is important to define what “physical activity” actually is. Researchers should also be aware that apart from significant benefits offered by EMA, there are various limitations to this approach which should be taken into consideration when attempting to conduct observations of PA.

## Figures and Tables

**Table 1:** Using EMA to evaluate PA among children and adolescents—a literature review compilation (2005–2009).

Authors (year)	Aim	Material	Timing	Activity/objective tool/EMA tool
Dunton et al. (2005) [9]	Using EMA to assess PA among adolescents	*n* = 526 ♂ 51% $\overset{-}{X}=14.47\pm 0.47$ Single survey	(i) Fall and spring each year (1998–2004); spaced approximately 6 months/4 days (Thursday–Sunday)/30 min intervals (±10) (ii) Three reminder signals/25–30 surveys	current activity—select from the list/A, HRm/e

Dunton et al. (2007) [19]	Using EMA to map the social and physical contexts of PA among adolescents	*n* = 502 ♀ 51% $\bar{X}=14.47\pm 0.47$ Surveyed 8 times during 4 years	(i) Fall and spring each year (1998–2004); spaced approximately 6 months/4 days (Thursday–Sunday)/30 min intervals (±10) (ii) 3 reminder signals/25–30 surveys	current activity—select from the list/A, HRm/e

Gorely et al. (2007) [20]	Using EMA to examine patterns of PA and SB across adolescents in the UK and to investigate if lifestyles of different groups differ on key explanatory variables	*n* = 1371 ♂ 521/♀ 850 $\bar{X}=14.7\pm 0.92$ Single survey	2 times: March–May and September–November (2000–2002); spaced approximately 6 months/3 weekdays, 1 weekend day/15 min intervals/44 surveys on weekdays and 68 on weekend days	current activity, with whom, and where—select from the list/p

Gorely et al. (2007) [21]	Using EMA to describe PA and SB in adolescent girls in the UK in their leisure time	♀ 923 $\bar{X}=14.7\pm 0.94$ (12.5–17.6 years) Single survey	2 times; spaced approximately 6 months/3 weekdays, 1 weekend day/15 min intervals/44 surveys on weekdays and 68 on weekend days	current activity, with whom, and where—select from the list/p

Biddle et al. (2009) [22]	Using EMA to describe prevalence of PA and SB in Scottish adolescents	*n* = 991 ♂ 385/♀ 606 $\bar{X}=14.1\pm 0.89$ Single survey	2 times: October-November 2002 and February-March 2003; spaced approximately 6 months/3 weekdays, 1 weekend day/15 min intervals/44 surveys on weekdays and 68 on weekend days	current activity—select from the list/p

Biddle et al. (2009) [23]	Investigate PA and SB in adolescents of different socioeconomic status in Central-Eastern European countries	*n* = 623 ♂ 247/♀ 376 $\bar{X}=15.5\pm 0.9$	3 weekdays, 1 weekend day/15 min intervals/44 surveys on weekdays and 68 on weekend days	current activity, with whom, and where—select from the list/p

Gorely et al. (2009) [24]	Investigate the relationships between family circumstance, PA, and SB among adolescents	*n* = 1171 ♂ 477/♀ 694 $\bar{X}=14.8\pm 0.86$ Single survey	2 times: March–May and September–November (2000–2002); spaced approximately 6 months/3 weekdays, 1 weekend day/15 min intervals/44 surveys on weekdays and 68 on weekend days	current activity, with whom, and where—select from the list/p

Gorely et al. (2009) [25]	Using EMA to describe PA and SB across adolescent boys in the UK in their leisure time	♂ 561 $\bar{X}=14.6\pm 0.89$ Single survey	2 times: March–May and September–November (2000–2002); spaced approximately 6 months/3 weekdays, 1 weekend day/15 min intervals/44 surveys on weekdays and 68 on weekend days	current activity, with whom, and where—select from the list/p

PA: physical activity; SB: sedentary behavior; EMA: ecological momentary assessment; *n*: number of subjects; ♂: males; ♀: females; $\bar{X}$: mean age [years] ± standard deviation; BMI: body mass index [kg/m^2^]; E: electronic diary/questionnaire on mobile phone with MyExperience software [16]; e: electronic diary in handheld computer; p: pencil and paper diary/questionnaire; p.c.: phone call; A: an accelerometer; HRm: a heart rate monitor.

**Table 2:** Using EMA to evaluate PA among children and adolescents—a literature review compilation (2010–2012).

Authors (year)	Aim	Material	Timing	Activity/objective tool/EMA tool
Rofey et al. (2010) [26]	Investigate feasibility of utilizing EMA in overweight adolescent girls and examine the relationships between EMA results, weight, and behavioral outcomes	♀ 20 11–19 years BMI = 39	(i) 3 extended weekends (Thursady–Monday) (ii) 2 times during weekdays and 4 times on weekends (iii) 14 phone calls	PA, current eating, affect, and social context/A/p.c.

Dunton et al. (2011) [27]	Testing the feasibility, acceptability, and validity of electronic EMA used to assess children's PA and SB	*n* = 121 ♂ 62/♀ 59 9–13 years	(i) 4 days (Friday–Monday) (ii) 3–7 surveys daily (iii) 20 prompts	current activity, with whom, and where—select from the list/A/E

Dunton et al. (2011) [28]	Using EMA to investigate level and experience of PA among children in their leisure time and social context	*n* = 120 ♂ 62/♀ 58 9–13 years	(i) 4 days (Friday–Monday) (ii) 3–7 surveys daily (iii) 20 prompts	current activity, with whom, social and physical context, current mood, and enjoyment—select from the list/A/E

Dunton et al. (2012) [29]	Using EMA to investigate whether children's perception corresponds with parents' perception of neighborhood characteristics, the children's level of PA in those settings, and their engagement in PA in leisure time	*n* = 108 ♂ 55% 9–13 years	(i) 2 times; spaced approximately 6 months (ii) 4 days (Friday–Monday) (iii) 20 prompts	current activity and physical context—select from the list/A/E

Dunton et al. (2012) [30]	Using EMA to determine whether children change the type of context when they engage in PA after a recent relocation to a SG	*n*_1_ = 46 ♂ 23/♀ 23 9–13 years *n*_2_ = 48 ♂ 26/♀ 22 9–13 years	(i) 2 times: from May–July to November-December in 2009; spaced approximately 6–12 months (ii) 4 days (Friday–Monday) (iii) 3–7 surveys daily (iv) 20 prompts	current activity, with whom, social and physical context, environmental perception, and enjoyment—select from the list/A/E

Dunton et al. (2012) [31]	Using EMA to describe where and with whom children engage in PA in their leisure time	*n* = 97 ♂ 53/♀ 44 9–13 years	(i) 4 days (Friday–Monday) (ii) 3–7 surveys daily (iii) 20 prompts	current activity, with whom, and social and physical context—select from the list/A/E

PA: physical activity; SB: sedentary behavior; EMA: ecological momentary assessment; *n*: number of subjects; *n*_1_: SG (a smart growth community); *n*_2_: control group ♂: males; ♀: females; $\bar{X}$: mean age [years] ± standard deviation; BMI: body mass index [kg/m^2^]; E: electronic diary/questionnaire on mobile phone with MyExperience software [16]; e: electronic diary in handheld computer; p: pencil and paper diary/questionnaire; p.c.: phone call; A: an accelerometer; HRm: a heart rate monitor.

**Table 3:** Using EMA to evaluate PA among adults—a literature review compilation (2007–2012).

Authors (year)	Aim	Material	Timing	Activity/objective tool/EMA tool
Vansteelandt et al. (2007) [32]	Using EMA to investigate associations between drive for thinness, emotional status, momentary urge to be physically active, and PA among adults with eating disorders	♀ 32 $\bar{X}=21.6\pm 6.7$ (15–37 years) BMI = 19.4 ± 4.4	(i) 7 days (ii) 9 times a day (iii) 63 surveys	urge to be physically active and PA, positive and negative emotional states/p

Dunton et al. (2009) [33]	Using EMA to investigate time-lag of submitted survey answers and to investigate effects of empirically supported social, cognitive, affective, and physiological factors supporting engagement in PA among adults	*n* = 23 ♀ 91% $\bar{X}=60.65\pm 8.22$ (50–76 years) BMI = 29.80 ± 6.29	(i) 2 weeks (ii) 4 times a day (iii) every 4 h (iv) 2 reminders every 10 min (v) 45 min to fill out a survey	current activity, how long (min), emotional status, and self-efficacy—select from the list/e

Kanning and Schlicht (2010) [34]	Analysis of association between daily activities and mood among healthy people	*n* = 13 ♂ 4/♀ 9 M = 56.5 (52–59 years)	(i) 10 weeks (from November 2006 to February 2007) (ii) 1–3 physically active episodes per day	current activity—unrestricted answer, mood/p

Rouse and Biddle (2010) [35]	Using EMA to assess PA and SB patterns of university students	*n* = 84 ♂ 46 ${\bar{X}}_{1}=20.2\pm 2.03$ ♀ 38 ${\bar{X}}_{2}=19.5\pm 1.15$	(i) 2 random days (1 weekday and 1 weekend day) (ii) 15 min intervals	current activity—unrestricted answer, with whom and where—select from the list/p

Bond et al. (2011) [36]	Using EMA to examine whether insufficient PA among bariatric surgery patients was due to infrequent PA intensions or inadequate follow through on PA intensions	*n* = 21 ♀ 81% $\bar{X}=48.5\pm 2.8$ ♀ 6 months after bariatric surgery	(i) 6 days (ii) in the morning and in the afternoon	PA—unrestricted answer, number of PA minutes (≥10 min), and barriers of PA/Talk Test/e

Dunton et al. (2012) [17]	Testing EMA protocol on mobile phone to assess adults' PA and SB	*n* = 110 ♂ 30/♀ 79 $\bar{X}=40.4\pm 9.74$ (27–73 years)	(i) 4 days (Saturday–Tuesday) (ii) 8 times per day (iii) 3 reminders every 5 min	current activity—select from the list/A/E

PA: physical activity; SB: sedentary behavior; EMA: ecological momentary assessment; *n*: number of subjects; ♂: males; ♀: females; $\bar{X}$: mean age [years] ± standard deviation; M: median; BMI: body mass index [kg/m^2^] ± standard deviation; E: electronic diary/questionnaire on mobile phone with MyExperience software [16]; e: electronic diary in handheld computer; p: pencil and paper diary/questionnaire; A: an accelerometer.
